# Geographical Classification of Italian Saffron (*Crocus sativus* L.) by Multi-Block Treatments of UV-Vis and IR Spectroscopic Data

**DOI:** 10.3390/molecules25102332

**Published:** 2020-05-16

**Authors:** Alessandra Biancolillo, Martina Foschi, Angelo Antonio D’Archivio

**Affiliations:** Department of Physical and Chemical Sciences, University of L’Aquila, Via Vetoio Coppito, 67100 L’Aquila, Italy; martina.foschi.mf@gmail.com (M.F.); angeloantonio.darchivio@univaq.it (A.A.D.)

**Keywords:** saffron, infrared, ultraviolet, classification, multi-block, data fusion, SO-PLS, SO-CovSel

## Abstract

One-hundred and fourteen samples of saffron harvested in four different Italian areas (three in Central Italy and one in the South) were investigated by IR and UV-Vis spectroscopies. Two different multi-block strategies, Sequential and Orthogonalized Partial Least Squares Linear Discriminant Analysis (SO-PLS-LDA) and Sequential and Orthogonalized Covariance Selection Linear Discriminant Analysis (SO-CovSel-LDA), were used to simultaneously handle the two data blocks and classify samples according to their geographical origin. Both multi-block approaches provided very satisfying results. Each model was investigated in order to understand which spectral variables contribute the most to the discrimination of samples, i.e., to the characterization of saffron harvested in the four different areas. The most accurate solution was provided by SO-PLS-LDA, which only misclassified three test samples over 31 (in external validation).

## 1. Introduction

Saffron, the dried stigma of Crocus sativus L., has been used since ancient times as a spice, food dye, or herbal medicinal. Crocins (a family of mono- or di-glycosyl esters of the polyene dicarboxylic acid crocetin), safranal (a monoterpene aldehyde), and picrocrocin (glycoside of safranal) are the saffron phytochemicals mainly responsible for its colour, aroma, and bitter taste, respectively [1,2]. Saffron is one of the foodstuffs most frequently subjected to commercial frauds because of high price (up to 25,000 €/kg) [3]. Since 1980 the International Organization for Standardization (ISO) establishes the methods for detecting extraneous substances in the spice and standard references for quality classification of commercial saffron [4,5]. UV-Vis spectroscopy is proposed by the above normative to estimate aroma, bitterness, and colouring strength based on the absorbance of an aqueous extract at 330, 257, and 440 nm, which depend on the contents of safranal, picrocrocin, and crocins, respectively. Reputation and commercial value of saffron is also linked to its geographical origin, since pedoclimatic factors and local know-how adopted in the cultivation of Crocus sativus L. and post-harvest drying process of the stigma have great impact on the final organoleptic properties of the spice. Therefore, geographical traceability is, together with quality assurance, a relevant issue to safeguard certified saffron from false labeling concerning the origin or reveal fraudulent mixing of certified saffron with low-quality products cultivated elsewhere. In this context, high-performance liquid chromatography [6,7,8], gas-chromatography [9,10], nuclear magnetic resonance spectroscopy [11], multi-elemental analysis of trace minerals [12,13], and stable isotopes of biogenic elements [14,15] have been applied to identify geographical markers and classify saffron according to the place of origin. Interestingly, Uv-visible spectroscopy on aqueous extracts, conventionally adopted to define the quality category of saffron according to ISO/TS 3632-2 specifications, also provides useful information regarding the geographical origin of this spice [16,17].

Infrared spectroscopy requires easy-to-use and cheap instrumentation and simple or no preliminary sample treatment when compared to most of the analytical techniques commonly utilized for tracing saffron. While near-infrared spectroscopy was often utilized to discriminate saffron produced in different countries [18,19,20], mid-infrared spectroscopy was mainly proposed for quality control or to detect specific adulterants in the spice [21,22,23,24,25]. A single application of mid-infrared spectroscopy in saffron geographical classification is described in literature based on our best knowledge [26]. In this study, discriminant analysis based on the spectra collected from powdered stigma provided poor discrimination of samples produced in Iran, Spain, Greece, and Italy, but classification performance improved when the spectra of the volatiles were used in discrimination, which required a preliminary ultrasound-assisted extraction of the volatile markers. In the present work, mid-infrared spectra were instead acquired from powdered saffron samples without any further manipulation and they were combined with UV-visible spectra of aqueous extracts by different data-fusion approaches to attempt a geographical classification of saffron produced in different, although relatively close, Italian areas. Eventually, two multi-block strategies, Sequential and Orthogonalized-Partial Least Squares Linear Discriminant Analysis (SO-PLS-LDA) [27] and Sequential and Orthogonalized-Covariance Selection Linear Discriminant Analysis (SO-CovSel-LDA) [28], were used to classify saffron samples according to their geographical origin in order to simultaneously handling both IR and UV-Vis data. These approaches were chosen, because discriminant analysis is a powerful tool, which is widely used in food analysis for tracing and/or authenticating agro-food products. The rationale behind the application of data fusion techniques is based on the consideration that it is much more efficient to handle multi-block data sets while using methods designed for it, rather than to inspect several individual models [29]. SO-PLS-LDA and SO-CovSel-LDA were adopted, because they performed very well in similar contexts [30,31,32,33,34]. Consequently, despite that this approach has never been reported before in the literature, the aim of the present work is to test whether UV-Vis and IR spectroscopies could be coupled with sequential multi-block strategies for tracing saffron.

## 2. Results

**Figure 1:**
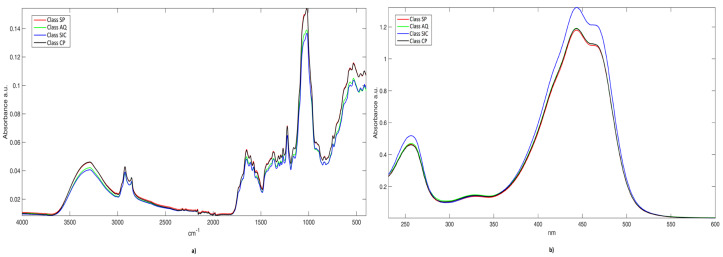
Mean raw spectra per class. **a**) IR signals; and, **b**) UV-Vis signals.

In Figure 1, the mean spectra per class are displayed. The peaks observed in the IR spectra (Figure 1a) are assigned to vibrational modes of specific saffron stigma constituents, according to [22,23,35,36] and the references therein. The broad band centered at about 3300 cm^−1^ is due to hydroxyl (O-H) stretching, while the peaks at 2916 and 2850 cm^−1^ correspond to C-H asymmetric and symmetric stretching. The bands in the range between 1800 and 1500 cm^−1^ are typical of vibrational mode of the carbonyl group and double bonds. The sharp signal at 1645 cm^−1^ results from the stretching modes of C=C and conjugated C=O (e.g., in picrocrocin), but amide I band of proteins also fall in this spectral region. The shoulders at higher wavenumbers (1740 and 1702 cm^−1^) are attributed to the C=O stretching in crocetin esters, aliphatic esters, and free carboxylic groups of crocetin and amino acids. Skeletal motions of the saffron constituents that are attributed to the CH/CH_2_, OH, C-C, C-O, and CCO moieties are responsible for the absorptions in the fingerprint region between 1500 and 1200 cm^−1^. The band at 1221 cm^−1^, in particular, is generated by the C-O stretching in the (C=O)-O group of crocetin esters. Most of the absorption bands in the 1200–700 cm^−1^ range result from vibrational modes of the sugar units and glycosidic linkages in polysaccharides or glycosyl moieties of crocins and flavonoids. The intense bands at 1051 and 1015 cm^−1^ are associated with C-O stretching vibrations in C-O-C groups of the sugar rings or glycosidic linkages, while the shoulder at 970 cm^−1^ originates from skeletal vibration modes of the glycosidic linkages. The bands in the range between 970 and 700 cm^−1^ can be attributed to C-H out-of-plane bending vibrations, while the absorptions below 500 cm^−1^ can take origin from skeletal breathing modes of oligo- and polysaccharides.

As for the UV-Vis spectra of aqueous saffron extracts (Figure 1b), the intense band that is centered at around 440 nm originates from the absorption of the polyene conjugated system of crocins and crocetin. The intensities of the secondary bands at 257 nm and 330 nm are conventionally attributed by ISO-3632 Technical Specifications to the contents of picrocrocin and safranal, respectively [4,5]. Nevertheless, absorptions of picrocrocin derivatives (with a maximum close to 250 nm) [2] and flavonoids, mainly kaempferol glycosides (in the range 265–349 nm) [37], also fall in the UV spectral region. In addition, the crocetin esters exhibit absorption secondary maxima at 250–260 nm (both *cis*- and *trans*-isomers) and 324–327 nm (only *cis*-crocins) [2,38].

Regardless of the class-membership, signals in both IR and UV-Vis spectra present similar shapes. IR spectra (Figure 1a) that are related to Class SP (red line) and Class CP (black line) are almost completely overlapped. Nevertheless, this is not surprising, because these saffron aliquots have been harvested in two adjoining areas. Additionally, UV-Vis spectra are very similar to each other; once again, samples belonging to Class SP, Class CP, and, to a lesser extent, Class AQ (green line), are overlaid; on the other hand, objects from Sicily (blue line) present a slight different absorbance intensity.

Prior to the analysis, samples were divided into a training and a test set by the Duplex algorithm in order to allow the external validation of the models [39]. Table 1 reports more details regarding the amount of calibration and validation objects, together with their class membership. SO-PLS-LDA and SO-CovSel-LDA models were calculated on the data organized as reported in the Table 1.

Prior to the creation of the calibration models, both classifiers require the optimization of model parameters, i.e., spectra preprocessing and number of features (latent variables (LVs) for SO-PLS, selected variables for SO-CovSel) to be extracted/selected. These were defined into a seven-fold cross-validation procedure involving only the training samples.

Six model parameters were tested on each block, i.e.,: Mean Centering, 1st Derivative (15 points window and second degree polynomial), 2nd Derivative (window of 15 points and third degree polynomial) [40], Standard Normal Variate (SNV) [41], SNV + 1st Derivative, and SNV + 2nd Derivative.

Testing six different combinations of pretreatments on two blocks leads to 36 diverse models due to the sequential nature of both the multi-block classifiers used (because all of the possible combinations between the two blocks differently preprocessed are calculated). For the sake of brevity and in order to not mislead the attention of the reader far from the focus of the present paper, the details about the optimization of model parameters are reported in Appendix A. In this section, only the outcomes of the best cross-validated models are reported. Partial Least Squares Discriminant Analysis also classified Uv-Vis and IR signals in order to assess whether the multi-block strategies actually represent an improvement with respect to the individual handling of the data. Appendix B reports the details on this latter approach.

### 2.1. SO-PLS-LDA Analysis

**Figure 2:**
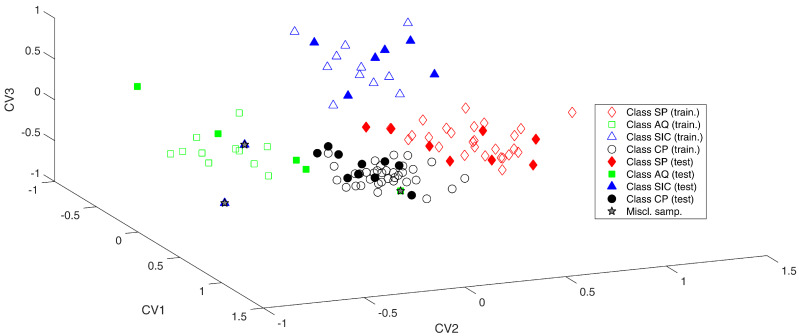
SO-PLS-LDA analysis. Samples projected onto the three canonical variate scores (CV).

As mentioned, diverse combinations of block-pretreatments were applied on data (see Appendix A); the SO-PLS-LDA model leading to the lowest error in cross-validation was the one built on IR preprocessed by SNV (extracting 3 LVs) and UV spectra pretreated by second derivative (extracting 13 LVs). The application of this model to the test set led to the classification results reported in Table 2 and displayed in Figure 2.

From the plot, it is evident that the four classes are well divided along the three canonical variates.

In particular, CV1 allows for discriminating Class AQ (green squares) and Class SIC (blue triangles) from the other two categories; in fact, Class AQ and SIC fall at negative values of this component, while Class SP (red diamond) and Class CP (black circles) are at positive ones. CV2 mainly discerns Class SP and Class SIC (at positive values) from Class CP and Class AQ (at negative CV-scores), finally CV3 allows for discriminating Class SIC (>0) from all of the other samples that present CV values minor than 0.

#### Interpretation of SO-PLS-LDA Models

##### VIP Analysis

**Figure 3:**
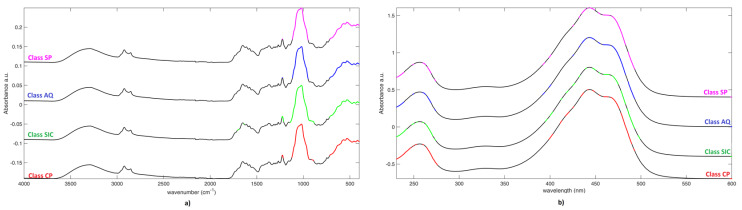
Variable Importance in Projection (VIP) analysis. **a**) on IR spectra; and, **b**) on UV spectra. Selected variables (VIP > 1) are colored.

Variable Importance in Projection (VIP) analysis was applied to the SO-PLS-LDA model in order to investigate which spectral variable contribute the most to the solution of the classification problem (following the embedded procedure described in [42]). As it is customarily done, variables presenting a VIP index higher than 1 were considered relevant; a graphical representation of the selected spectral features is reported in Figure 3 (mean spectra were offset to make them visible). When considering the IR data block (Figure 3a), regardless the class-membership, the peak at 1018 cm^−1^ and variables between 761 cm^−1^ and (about) 500 cm^−1^ were always selected. Additionally, for all categories, the spectral variable around 1224 cm^−1^ are also relevant, but a wider/narrow range of feature is selected, depending on the class. According to the interpretation of the IR spectra presented previously, the inter-class differentiation seems to be related with the differences in the intensities of absorptions due to crocetin esters and sugars. Eventually, the main difference among the four categories is represented by the fact that, for samples belonging to Class SIC, also few variables around 1699 cm^−1^ and the variable at 1637 cm^−1^ are relevant (while they are not selected in the other categories), which suggests the additional role of the picrocrocin content in the discrimination of this saffron class.

VIP analysis on the UV-Vis-block provided similar results among the diverse categories. In fact, it generally pointed out variables between 390 nm and 500 nm, due to the absorption of crocins, and those between 230nm and 280 nm, being mainly dependent on the content of picrocrocin and flavonoids. Additionally, in this case, objects appertaining to Class SIC, where a more parsimonious selection was made, but always in the same two ranges, provide the most different outcome.

### 2.2. SO-CovSel-LDA Analysis

As described for SO-PLS-LDA, also for SO-CovSel-LDA analysis, diverse pretreatments (and their combinations) were tested on data (see Appendix A). Additionally, in this case, the optimal model (i.e., the one leading to lowest classification error in cross-validation) was the one calculated on IR spectra preprocessed by SNV and UV-Vis signals pretreated by second derivative. In total, 10 spectral variables were selected by CovSel, one in the IR block and nine from the Uv-Vis spectra. Eventually, the model was used to predict test samples, providing the results that are reported in Table 3.

#### Interpretation of SO-CovSel-LDA Models

**Figure 4:**
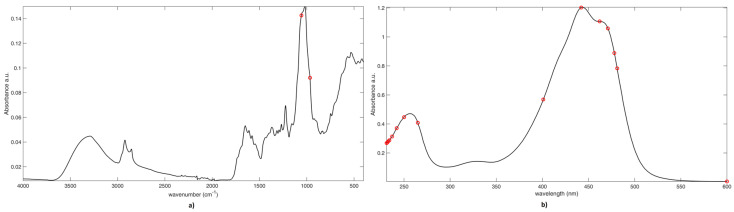
SO-CovSel-LDA Analysis. Graphical representation of variables selected on **a**) IR block; and, **b**) UV block. Legend: Black line: Mean Spectrum. Red Circles: Selected variables.

Despite being much more parsimonious, the selection made by CovSel is in strong agreement with the one provided by VIP analysis (shown in Figure 4). In fact, the spectral IR variables selected are those at 1057cm^−1^ and 966 cm^−1^ attributable to typical vibrations of the sugar moieties or glycosidic linkages. On the other hand, the 12 variables selected by CovSel on the UV-Vis block are between 231 nm and 264 nm, and some between 400 nm and 500 nm (i.e., variables at 400 nm, 442 nm, 461 nm, 470 nm, 477 nm, and 481 nm) plus 600 nm. These results confirm the discriminant role of picrocrocin, flavonoids, and crocins. It must be noted that the individual crocins detected in saffron, differing in the cis or trans isomeric form of crocetin and the kind of sugar(s) (mostly glucoside, gentiobioside, triglucoside, and neapolitanoside), present some differences in the UV-vis band centered at 440 nm concerning the position of the two absorption maxima and their relative intensities [2,38]. Based on the role of the fine details of the band centered at 440 nm, saffron geographical discrimination seems to be related with the relative content of the different crocins in the spice rather than their global concentration.

## 3. Materials and Methods

### 3.1. Saffron Sample Set

One hundred and fourteen samples of saffron were available for the analysis; IR and UV-Vis spectrscopies investigated aliquots of this spice (applying the procedures exposed below in Section 3.2). The samples were harvested in four different Italian geographical areas: Spoleto (Umbria region, Central Italy), L’Aquila (Abruzzo region, Central Italy), Sicily (South Italy), and Città della Pieve (Umbria region, Central Italy). Of these, two towns (Spoleto and Città della Pieve) belong to the same region (Umbria) and they are quite close (around 80 km) to one and another. L’Aquila is in a different region (Abruzzo) in Central Italy, and it is around 100 km far from Spoleto, and 180 km from Città della Pieve. These three geografical areas present comparable pedoclimatic conditions. Sicily is an island in South Italy; consequently, it presents peculiar climatic and geological conditions, quite different from those encountered in the other two mentioned peninsular regions.

**Figure 5:**
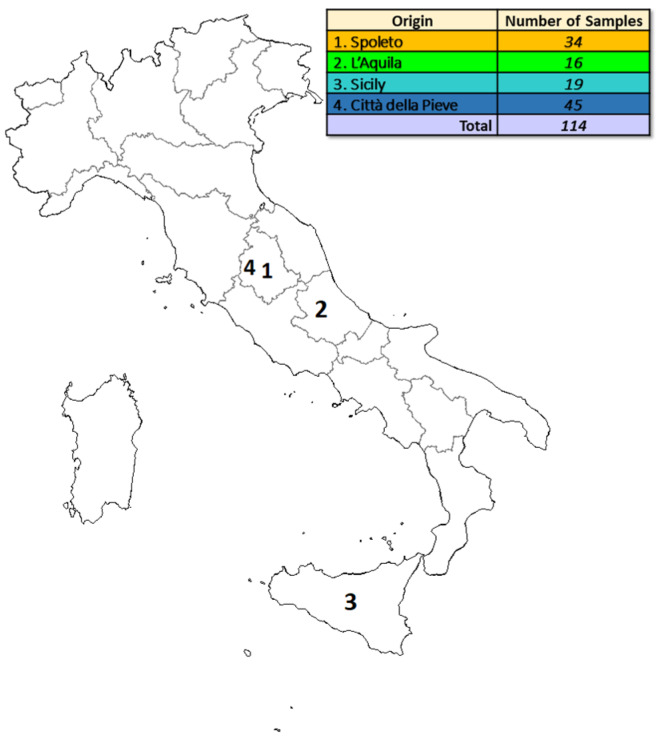
Details about the origin and the numerosity of the analyzed samples.

Figure 5 reports more details about the origin and the number of samples.

### 3.2. Instrumental Apparatus and Data Collection Procedure

Regardless of the analytical technique used, all of the available spectra were exported and elaborated in MATLAB (The Mathworks, Natick, MA; version 2015b) while using in-house functions.

#### 3.2.1. FT-IR Spectroscopy

The infrared spectra of the saffron powder, obtained by freshly grinding the stigma with a mortar, were recorded on a PerkinElmer Spectrum Two™ (PerkinElmer, Waltham, MA, USA) FTIR spectrometer consisting of a deuterated triglycine sulfate detector and a PerkinElmer Universal Attenuated Total Reflectance (uATR) accessory equipped with a single bounce diamond crystal. A consistent force was applied on the sample while using the pressure monitoring system integrated with the instrument to maximize the spectrum intensity. Each spectrum was registered from 4000 cm^−1^ to 400 cm^−1^ with a 1 cm^−1^ instrumental resolution, and ten scans were averaged per spectral replicate. The background was collected with the crystal that was exposed to the air.

#### 3.2.2. UV-Vis Spectroscopy

Sample preparation was carried out according to the procedure that was suggested by ISO-3632 [5], but saffron and solvent amounts were proportionally reduced. About 50 mg of saffron stigma were gently ground in a mortar. 10 mg of ground sample were suspended in 20 mL volumetric flask that was filled with 18 mL of distilled water; the suspension was kept under magnetic stirring for 1 h in the dark; and finally, diluted to 20 mL. The spectrophotometric measurement was carried out on a suitable aliquot of aqueous extract after a 10-fold dilution and filtration on a 0.45 μm Whatman Spartan 13/0.2 RC (Whatman, GE Healthcare Life Sciences, Little Chalfont, UK) cellulose filter. The UV-vis spectra were acquired in the 200–700 nm range with a Cary 50 Probe (Agilent Technologies, Santa Clara, CA, USA) spectrophotometer using a 1 cm pathway quartz cuvette and pure water for blank correction. The spectra were recorded with a 1 nm resolution.

### 3.3. Multi-Block Classifiers

#### 3.3.1. Sequential and Orthogonalized-PLS Linear Discriminant Analysis (SO-PLS-LDA)

Sequential and Orthogonalized Partial Least Squares (SO-PLS) [43] is a multi-block method developed to solve regression problems that were recently extended to the classification field by combination with Linear Discriminant Analysis (LDA [44]). The resulting method, SO-PLS-LDA [27,45], is a multi-block classifier suitable in different contexts.

Taking into account a two data block-case, X_(N × L)_ and Z_(N × M)_, the algorithm is quite simple, and it can be summarized by the four step below:

1. X is used to predict the Y response by means of PLS.
2. Z is orthogonalized with respect to the X-scores estimated in step 1, obtaining Z_Orth._
3. Residuals from step 1. are fitted to Z_Orth_ by PLS.
4. The predictive model is calculated by summing up results from step 1 and step 3, obtaining the predicted Y.
5. LDA is applied on the predicted Y (or on the concatenated X- and Z-scores).

SO-PLS-LDA models were calculated using in-house functions for Matlab (freely downloadable at https://www.chem.uniroma1.it/romechemometrics/research/algorithms/so-pls/).

#### 3.3.2. Sequential and Orthogonalized-Covariance Selection (SO-CovSel)

Sequential and Orthogonalized-Covariance Selection (SO-CovSel [28]) is a sequential multi-block approach as SO-PLS. Plainly, the algorithm of SO-CovSel is the same as SO-PLS, but the feature reduction operated by PLS is performed by a variable selection approach called Covariance Selection (CovSel) [46]. Obviously, this leads to further slight differences in their algorithms.

When considering the same data blocks that are involved in Section 3.3.1 for SO-PLS, the building process of an SO-CovSel model can be summarized, as follows:

1. Covariance Selection is used to select a fixed amount of variables from the X-block; eventually, the reduced X-block is obtained (X_Sel_).
2. X_Sel_ is used to estimate the Y by ordinary least square regression.
3. Z is orthogonalized with respect to X_Sel_, obtaining Z_Orth_.
4. Z_Orth_-variables are selected by Covariance Selection (and organized in a unique matrix Z_Orth,Sel_).
5. Z_Orth,Sel_. is used to estimate the Y by ordinary least square regression
6. The final predictive model is obtained combining results from step 2 and step 4, obtaining the predicted Y.
7. LDA is applied on the predicted Y.

SO-CovSel-LDA models were calculated while using in-house functions for Matlab (freely downloadable at: https://www.chem.uniroma1.it/romechemometrics/research/algorithms/so-covsel/)

## 4. Conclusions

The aim of the present study was to discriminate Italian saffron samples that were harvested in four different geographical areas on the basis of their IR and UV-Vis profiles collected from powdered stigma and aqueous extracts, respectively. Two multi-block strategies have been exploited in order to achieve this goal: SO-PLS-LDA and SO-CovSel-LDA. In general, both approaches provided satisfactory results, demonstrating to perform better than the PLS-DA analysis of the individual blocks (see Appendix B). The most accurate results were provided by SO-PLS-LDA, which only misclassified three samples over 31 of the external test set. A further inspection of the results, based on the comparison of the outcomes of SO-PLS-LDA and SO-CovSel-LDA, has unveiled the two models that were misclassified the same three samples (i.e., all of the objects misclassified by SO-PLS-LDA); the agreement between the two approaches suggests that these samples present peculiar characteristics different from the other saffron belonging to their category. Among these objects, one belongs to Class AQ, and the others to Class SIC. Nevertheless, it is not completely surprising that samples belonging to this latter category are confused. In fact, contrarily to all other samples, which originate from a very restricted area, circumstantiated to the borders of a single town, samples from Sicily have been harvested in a wider area. As a consequence, this difference confers to Class SIC, a wider inner-class variance, which makes the classification of its objects a bit more complex.

## Figures and Tables

**Table 1:** Organization of samples into training and test sets.

	Training (N. Samples)	Test (N. Samples)	Total (N. Samples)
Class Spoleto (SP)	26	8	34
Class Aquila (AQ)	11	5	16
Class Sicily (SIC)	11	8	19
Class Città della Pieve (CP)	35	10	45
Total	83	31	114

**Table 2:** Sequential and Orthogonalized Partial Least Squares Linear Discriminant Analysis (SO-PLS-LDA) analysis: External Validation. Correct classification rates (%) and number of misclassified test samples.

Predictions (on the Test Set)							
Class SP		Class AQ		Class SIC		Class CP	
Class. Rate (%)	Misclass. samples	Class. Rate (%)	Misclass. samples	Class. Rate (%)	Misclass. samples	Class. Rate (%)	Misclass. samples
100.0	0	80.0	1	75.0	2	100.0	0

**Table 3:** SO-CovSel-LDA analysis: External Validation. Correct classification rates (%) and number of misclassified test samples.

Predictions (on the Test Set)							
Class SP		Class AQ		Class SIC		Class CP	
Class. Rate (%)	Misclass. samples	Class. Rate (%)	Misclass. samples	Class. Rate (%)	Misclass. samples	Class. Rate (%)	Misclass. samples
100.0	0	80.0	1	62.5	3	100.0	0

**Figure A1:**
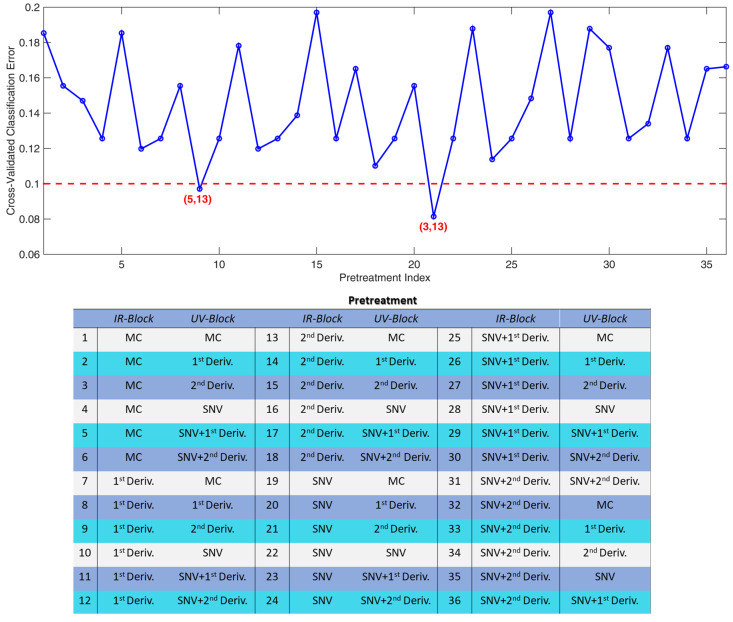
SO-PLS-LDA. Cross-validated classification errors. Numbers in brackets represent the number of LV extracted by the IR and the UV-Vis blocks, respectively. The dashed line is not a cut-off value, it is meant to help the appreciation of the local minima. In the table, “MC” stands for “mean centering” and “Deriv.” for “derivative”.

**Figure A2:**
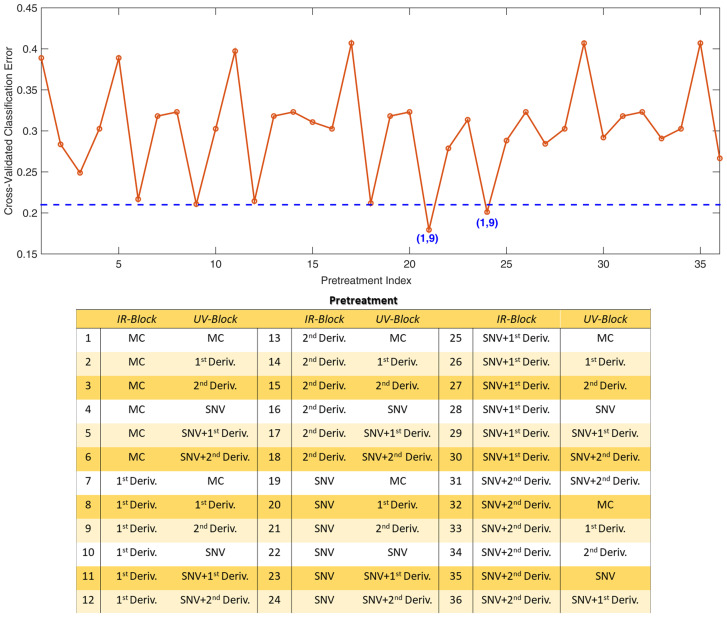
SO-CovSel-LDA Cross-validated Classification errors. Numbers in brackets represent the number of variables selected in the IR and the UV-Vis blocks, respectively. The dashed line is not a cut-off value, it is meant to help the appreciation of the local minima. In the table, “MC” stands for “mean centering” and “Deriv.” for “derivative”.

## Appendix A. Definition of the Optimal Complexity in the Multi-Block Models

Regardless the classifier used, different combinations of pretreatments were tested on data, and then, the optimal model was defined into a cross-validation procedure.

For both SO-PLS-LDA and SO-CovSel-LDA 36 diverse models were built. The cross-validated errors are displayed in Figure A1 and Figure A2, respectively.

In the plots, the errors are reported as function of the preprocessing approaches applied, listed on the right side of the graphs. The numbers reported in the lists represent the pretreatment indices.

In Figure A1 the number in bracket below each point represents the number of LVs selected (for the IR, and the UV block, respectively); similarly, in Figure A2, the numbers refer to the selected variables (for the IR, and the UV block, respectively). From the plots it is evident different models provided similar cross-validated classification errors: the most parsimonious solutions (in terms of number of components) were chosen.

From Figure A1, it is clear two SO-PLS-LDA models provided similar, low classification errors. The chosen model is the one built using the most parsimonious set of latent variables, i.e., the one using 16 LVs in total (the other one required 18 LVs). The definition of model parameters for SO-CovSel-LDA was defined in a similar way; the best models presented the same optimal complexity: the model providing the lowest classification error was chosen.

## Appendix B

PLS-DA analysis was run on the individual IR and UV-Vis signals. Data were preprocessed by the same pretreatment used in the multi-block strategies. The outcome of the PLS-DA models calculated on IR data are reported in Table A1; the results obtained when PLS-DA is applied on UV-Vis spectra are shown in Table A2.

**Table A1:** PLS-DA analysis on IR data: Correct classification rates (%) for calibration (CV) and validation (test set).

IR				
	Class SP	Class AQ	Class SIC	Class CP
Calibration (CV)	Class. Rate (%)	Class. Rate (%)	Class. Rate (%)	Class. Rate (%)
	57.7	72.7	54.5	40.0
Prediction (on the test set)	75.0	80.0	25.0	60.0

**Table A2:** PLS-DA analysis: Correct classification rates (%) for calibration (CV) and validation (test set).

UV-Vis				
	Class SP	Class AQ	Class SIC	Class CP
Calibration	Class. Rate (%)	Class. Rate (%)	Class. Rate (%)	Class. Rate (%)
	62.5	40.0	75.0	90.0
Prediction	91.3	84.6	87.5	72.9
